# Enhancing passive surveillance for African swine fever detection on U.S. swine farms

**DOI:** 10.3389/fvets.2022.1080150

**Published:** 2022-12-02

**Authors:** Rachel Schambow, Yoder Colin, Wright Dave, Daniella N. Schettino, Andres M. Perez

**Affiliations:** ^1^Center for Animal Health and Food Safety, University of Minnesota, Saint Paul, MN, United States; ^2^Department of Veterinary Population Medicine, College of Veterinary Medicine, University of Minnesota, Saint Paul, MN, United States; ^3^Private Veterinarian, Buffalo, MN, United States; ^4^Instituto de Defesa Agropecuária do Estado de Mato Grosso (INDEA/MT), Cuiabá, Mato Grosso, Brazil

**Keywords:** African swine fever, disease surveillance, enhanced passive surveillance, foreign animal disease, pig, participatory

## Abstract

As the threat of African swine fever (ASF) introduction into new areas continues, animal health officials and epidemiologists need novel tools for early detection and surveillance. Passive surveillance from swine producers and veterinarians is critical to identify cases, especially the first introduction. Enhanced passive surveillance (EPS) protocols are needed that maximize temporal sensitivity for early ASF detection yet are easily implemented. Regularly collected production and disease data on swine farms may pose an opportunity for developing EPS protocols. To better understand the types of data regularly collected on swine farms and on-farm disease surveillance, a questionnaire was distributed in summer 2022 across multiple channels to MN swine producers. Thirty responses were received that indicated the majority of farms collect various types of disease information and conduct routine diagnostic testing for endemic swine diseases. Following this, a focus group discussion was held at the 2022 Leman Swine Conference where private and public stakeholders discussed the potential value of EPS, opportunities for collaboration, and challenges. The reported value of EPS varied by stakeholder group, but generally participants felt that for swine producers and packers, EPS would help identify abnormal disease occurrences. Many opportunities were identified for collaboration with ongoing industry initiatives and swine management software. Challenges included maintaining motivation for participation in ASF-free areas, labor, data sharing issues, and the cost of diagnostic testing. These highlight important issues to address, and future collaborations can help in the development of practical, fit-for-purpose, and valuable EPS protocols for ASF detection in the swine industry.

## Introduction

Foreign animal diseases (FADs), such as African swine fever (ASF), cause significant global economic and health burden to the swine industry. ASF is caused by the ASF virus (ASFV), a large, enveloped DNA arbovirus that only affects swine, including domestic pigs and wild boar (1, 2). No treatment or readily approved and available vaccine exist to help mitigate its impact, so identification of infected herds followed by depopulation is primarily used to control disease spread and for eradication. In addition to trade restrictions imposed for ASF-infected countries, infection with the ASFV may cause devastatingly high mortalities in affected farms and wide-scale losses due to culling. In recent years, ASF has spread throughout Africa, Asia, Europe, and to the island of Hispaniola containing Haiti and the Dominican Republic in the Caribbean (3). The ongoing ASF global spread has raised serious concerns of a potential disease introduction into the United States (U.S.). An introduction into the U.S. would immediately halt all swine trade and exports and lead to widespread losses of pigs, with recovery estimated at costing $50 billion over 10 years (4). To prevent such a catastrophic scenario, animal health officials rely on strategies of detection and depopulation to identify, contain, and eradicate ASF outbreaks (5).

Global ASF spread highlights the importance of disease surveillance even in apparently disease-free areas. The availability of high-quality diagnostic tests with targeted active surveillance has substantially decreased the time to confirm suspect ASF cases to hours after sample collection (6–8). However, these systems do not decrease the time for swine producers and veterinarians to identify suspect cases on farms, and the time to identify an initial suspect after the first introduction into a country is highly uncertain (9–11). Achieving high coverage of the population is often difficult and expensive with active surveillance. Passive surveillance of animal populations, whereby disease reporting is initiated directly by animal observers such as farmers or primary veterinarians, is highly valuable for monitoring otherwise unreached populations and for increasing the overall sensitivity of a surveillance system. Passive reporting has been especially critical for initial detections of ASF (12–14), and enhancing these strategies will likely be more effective at early ASF detection. Regularly collected information from swine production systems may help create the foundations for a constant flow of data and associated algorithms monitoring for signals that could indicate a FAD such as ASF. In recent years, many groups have explored methods of syndromic surveillance for diseases and pathogens like ASFV or Porcine Reproductive and Respiratory Syndrome virus (PRRSV) with swine data or with technologies like activity monitors or cameras (15–17). These showed some potential success for decreasing detection time for swine pathogens, and collectively, demonstrate that disease surveillance through swine data monitoring may be possible if appropriate data are available.

Practical considerations of the U.S. swine industry preclude an easily implementable, national surveillance system for ASF and other FADs. In the absence of an animal health emergency that justifies governmental intervention, data sharing with animal health officials is not mandatory for U.S. swine producers, and consequently, accessibility to the data necessary to implement widespread surveillance is limited. In recent years, some voluntary initiatives to manage and control domestic infectious swine diseases in the U.S. have had high participation and success. For example, the Morrison Swine Health Monitoring Program (MSHMP) is a national control project started in 2011 to better understand PRRSV epidemiology in the U.S. (18). The program relies on voluntary participation and has significantly helped in the understanding and control of PRRSV (19, 20). Its success has led to the extension of the program to monitoring of other swine pathogens (18, 21).

To develop an enhanced passive surveillance protocol (EPS) for ASF, a better understanding of the current state of swine data capacity is necessary. Objectives of the work here were to characterize regularly collected swine data and disease surveillance activities on U.S. swine farms, explore how these activities could be used for ASF surveillance, and identify how ongoing swine industry technologies and initiatives for disease preparedness could be collaborative to improve ASF surveillance. We explored through a combination of mixed qualitative methods the types of data and management systems used by swine farmers in Minnesota, one of the top swine producing states in the U.S. We later convened a workshop of researchers, government officials, producers, veterinarians, and management software representatives to discuss the potential for EPS implementation on swine farms. Results collected and assessed here will help to identify next steps and priorities for EPS development and opportunities for collaborations between ongoing ASF surveillance efforts.

## Analytical approach

To understand the current state of swine data collection and disease surveillance and to characterize the potential for EPS, two stages of data collection were designed following a modified Delphi approach (22). First, an anonymous questionnaire was developed in Qualtrics to characterize the types of data collected on swine farms and practices for disease surveillance and to give a baseline understanding that would inform future in-person discussions. The questionnaire is available in full in Supplementary File 1. Generally, questions asked about the respondent's swine operation, participation in industry initiatives for FAD-preparedness, the type of software or method used to collect swine farm data, the type and frequency of disease, production, and breeding data collection, and on-farm disease surveillance including diseases routinely tested for, routinely collected specimens, necropsy protocols, and disease investigation triggers. At the end, respondents were able to indicate their interest in participating in a future EPS study through an additional one-question Qualtrics questionnaire, to maintain their anonymity to the first questionnaire. The questionnaire was beta-tested with three purposely-selected MN swine producers or veterinarians for feedback on clarity and structure. A targeted list of individuals was not selected in advance to receive the questionnaire; instead, it was openly distributed through email addresses available from Secure Pork Supply (SPS) program activities in MN and through advertisements in the MN Board of Animal Health, University of MN Swine Extension, and MN Pork Board newsletters. These channels were chosen through discussions with a former swine veterinarian and SPS program leader because they represent main modes of communication and education to MN swine producers and would likely reach a wide audience. Briefly, the SPS program is a voluntary, industry-led initiative promoting the development of on-farm biosecurity plans (23). The University of MN Swine Extension is an educational service for sharing information with swine producers (24). The MN Board of Animal Health is the government agency managing animal health issues and rules within Minnesota (25). Finally, the MN Pork Board is an industry-led board with USDA oversight that supports swine producers within the state and oversees Pork Checkoff activities (26). The questionnaire was kept open from June to August 2022.

The information collected from the questionnaire was used to guide the development of in-person activities at one of the most important swine health outreach events annually organized in the U.S., referred to as the Allen D. Leman Swine Conference in St. Paul, MN. The Leman Swine Conference is an international conference that draws one of the largest groups of academic and professional attendees from across the swine industry to share current swine research. This participatory approach, whereby participants of an ongoing program were involved to help inform research activities, has previously been used in veterinary epidemiology to support the development of risk assessments for foot-and-mouth disease (27). First, an open workshop introducing the EPS approach and related approaches was organized. Talks were presented from USDA, academia, and private industry that focused on analytical tools to support ASF preparedness and surveillance. The objective of that initial open activity was to familiarize the audience with key concepts and ideas to inform the discussion. The following day, a focus group was organized to prompt the review and discussion of collected answers from the initial questionnaire and the potential for EPS protocols on swine farms (28). In total, 74 individuals were invited to participate in the focus group discussion. These individuals represented research/academia, private swine software companies, USDA, NPB, AASV, primary swine veterinary clinics, and private swine farms and companies. Approximately one quarter of the invitees (*n* = 19) and 4 moderators attended the discussion, which was organized following a world-café format (29). The participants were given a brief introduction of disease surveillance and EPS, which summarized the presentations from the previous day, and a summary of the questionnaire results. Participants were then given the choice to join one of four topics:

1. Do you see value for EPS for the industry (depending on the epidemiological conditions of the country) for FAD detection?

2. What is needed for swine data on farms for a successful EPS system?

3. What are opportunities for collaboration for FAD surveillance and preparedness?

4. What are challenges for EPS implementation?

Each table had approximately 30 minutes to discuss their assigned question as a small group, which was coordinated by a moderator, from the authorial team, to facilitate and record the discussion. Conclusions were then presented for the whole group to discuss. Each moderators' recorded notes were later reviewed and summarized.

## Results

### Questionnaire summary

Thirty questionnaire responses were received, of which 25 were fully completed and 5 were partially completed. All farm types in the questionnaire were represented, and the majority were sow farms (Table 1). Sixteen respondents reported having multiple production sites (ranging from 1 to 27). Twenty-nine had veterinary access, of which 21 had a veterinarian regularly visit while only 8 visited for specific concerns only (No response = 1). Twelve were enrolled in National Pork Board's online contact tracing platform, though 14 were unfamiliar with it. Three were familiar but unenrolled (no response = 1). Conversely, 23 had a SPS biosecurity plan (no response = 1) and only one was not aware of the program. Disease events were primarily recognized by farm staff (*n* = 19) or managers/owners (*n* = 7, no response = 4), and none reported by their veterinarian. Nineteen respondents thought they would recognize signs of a FAD, but five were unsure (no response = 4). Many different factors were reported to trigger further disease investigation, including increased mortality or morbidity (*n* = 22), changes in feed (*n* = 15) or water consumption (*n* = 14), or a “gut feeling” (*n* = 14, no response = 4). Fifteen felt they could detect a drop in feed consumption within a day, while others estimated within hours (*n* = 3) or a week (*n* = 7, no response = 5). Software usage was high (*n* = 25, no response = 1). Some used multiple types of software, and only four farms, of which three were finisher farms, used none. All data was primarily collected by farm staff either through hand-written records (*n* = 15) or digital handheld technology (*n* = 12, no response = 1, owner/off-farm staff = 2). Nineteen completed this on their own, though some used a management company (*n* = 6) and/or their veterinarian (*n* = 3, no response = 3).

**Table 1:** Characteristics of survey respondents.

**Farm type**	**Number of respondents**
Boar stud	3
Farrow-to-finish	3
Finisher	8
Genetic multiplier	1
Gilt Development Unit	1
Nursery	4
Sow	9
Other: Isowean-to-finish	1

**Figure 1:**
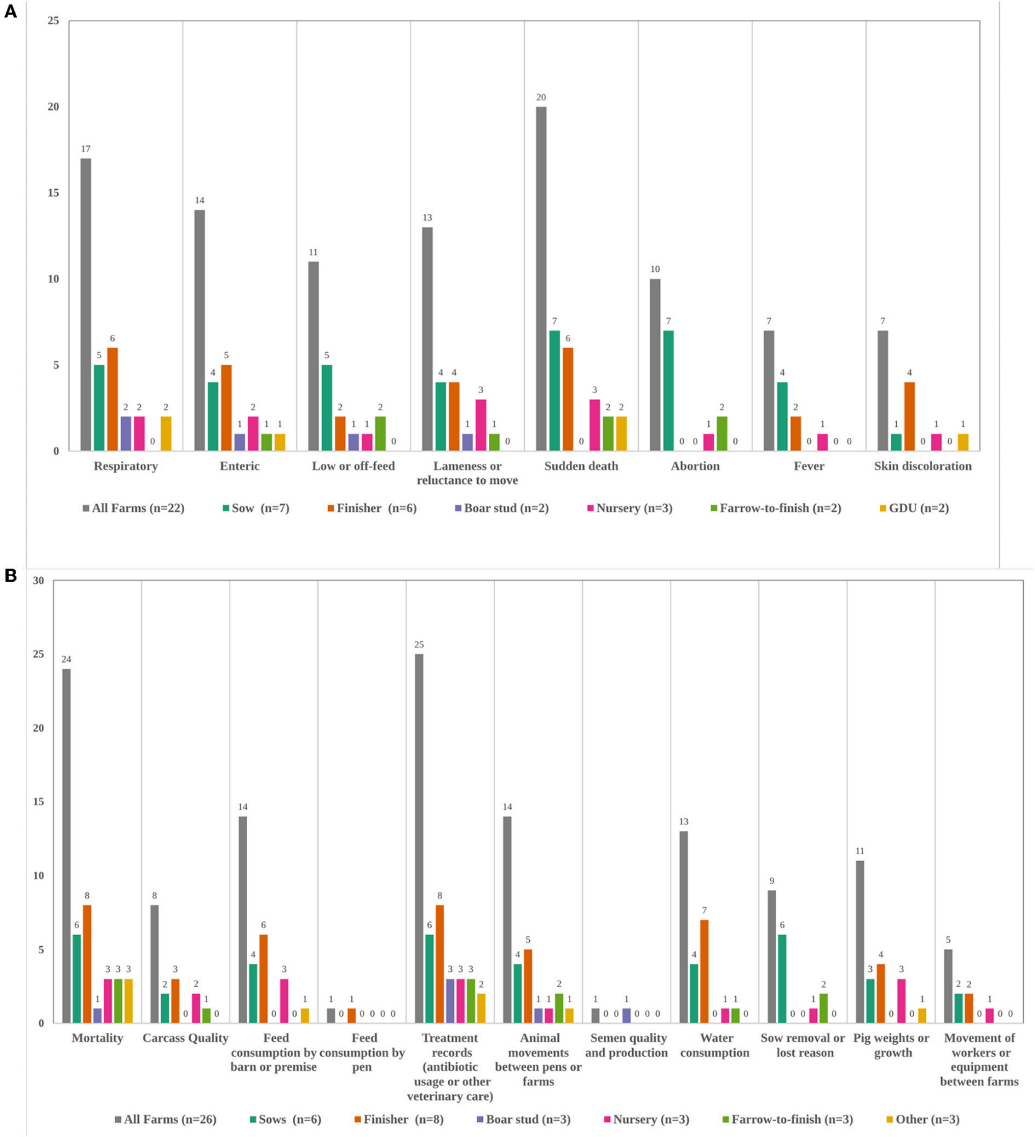
**(A,B)** Types of disease events **(A)** and production data **(B)** recorded on farms, by total and farm type. Total farm number for disease events **(A)** is out of those that answered “Yes” to recording any type of disease information and selected at least one disease event (*n* = 22, no response = 1). No farms reported huddling. For production data **(B)** “other” included 1 gilt development unit, 1 isowean to finish farm, and 1 genetic multiplier. Three sow farms and one nursery farm did not specify what type of production information they recorded. GDU, Gilt Development Unit.

Disease event information was collected on 23 farms (no response = 1). On sow or boar stud farms, records were always recorded for individual animals, otherwise group records were more common. The most commonly recorded events were sudden death, respiratory, and enteric signs, but this varied some by farm type (Figure 1A). Of these 23 farms, 16 (no response = 2) also recorded a confirmed or presumptive pathogen. Fourteen recorded the occurrence of observed disease events daily, and three recorded multiple days a week or weekly (no response = 2). Four had no set schedule of recording observed events. Production records were collected on all farms (Figure 1B, no response = 4). The most commonly recorded information was treatment records (antibiotic usage or other veterinary care) and mortality, while the least recorded was movement of workers, feed consumption by pen, and semen quality. Breeding records were collected on 6 sow farms, 3 farrow-to-finish farms, and 1 nursery farm. These all included breeding dates, pregnancy check results, rebreeding events, abortion dates, stillbirths, and mummies. Two farms also recorded abortion cause.

PRRSV (*n* = 21) and porcine epidemic diarrhea virus (*n* = 17) were the most routinely tested domestic diseases, followed by transmissible gastroenteritis virus (*n* = 14), porcine deltacoronavirus (*n* = 11), influenza (*n* = 9), Mycoplasma hyopneumoniae (*n* = 7) and porcine circovirus type 2 (*n* = 3). Five farms did no routine testing (no response = 4). Oral fluids (*n* = 21) and blood (*n* = 20) were the most commonly collected specimens. The collection of blood may be for serum collection, but that was not specified or distinguished here. Only 3 recorded diagnostic test results into their management software (no response = 4). Fourteen performed routine necropsies by farm staff or veterinarians, though 12 didn't (no response = 4), and those that did only performed them infrequently or during large-scale outbreaks. Nineteen farms felt they would be comfortable necropsying pigs themselves and collecting samples (no response = 4), and of these many felt comfortable collecting spleen (*n* = 14), tonsils (*n* = 9), or superficial lymph nodes (*n* = 8).

### World-café discussion findings

Discussion covered the potential value and benefits of EPS for the swine industry, data needs for surveillance, opportunities for collaboration, and challenges. The reported value of EPS was highly different by stakeholder. For producers and packers, EPS protocols could be valuable to differentiate domestic and foreign diseases and identify concerning disease trends. They could support ASF case definitions and help identify suspect pigs to target for sampling. For small producers in particular, EPS systems could support awareness of ASF and serve as surveillance tools in resource-limited situations. Prior to an ASF outbreak, these activities would support communication about disease events between farm employees and management. Participants also suggested that collected data could be used to forecast domestic disease outbreaks. With increased usage across many producers and sites, data could potentially be used to create regional risk maps for disease outbreaks that could be informative to swine producers. Veterinarians could increase business from helping their producers implement and maintain these protocols. EPS could be beneficial for government and veterinary diagnostic labs by prioritizing limited testing resources to suspect farms, and by incentivizing development of multiplex diagnostic tests to complement domestic disease surveillance. Participants felt that value to wholesalers and resellers would be limited as they would likely adjust what they sell according to the market trends. Potential incentives for participation included improving the detection of endemic diseases, such as PRRSV, or financial incentives like decreased insurance rates or quicker return to shipping animals in the event of an outbreak.

Many data needs were identified. Daily data collection at the pen or barn-level would be ideal and provide sufficient opportunity for early detection of highly-virulent ASF strains. Weekly collection was suggested as viable for detecting moderately-virulent ASF strains, but premise-level data wouldn't be sensitive enough for early detection. Data would need to be automatically or quickly uploaded to a centralized source for analysis. Participants were concerned that if data were collected *via* hand-written records, it would take up to a week for entry into a database, and the resulting time lag would be too great. Participants also highly emphasized the need for a simple system that could be used daily by on-farm workers with minimal training, especially because farm owners or managers may only visit a given site on a weekly basis. Ideally, data would be collected through mobile apps within software programs producers already own. Easily understood questions, such as a “yes/no” format or checklist, in multiple languages would facilitate collection and increase data quality. Offline software capability would be important because many farms in the U.S. have limited or no access to Internet or cellular services. Finally, standardized data fields would allow for better communication between software and analysis.

Many potential opportunities were reported. Swine management software could be modified for collecting relevant data, assuming a standardized design with producer support were developed. Industry initiatives could also support EPS. The U.S. Swine Health Improvement Plan (U.S. SHIP) is a USDA-sponsored initiative to improve swine health, biosecurity, traceability, and disease surveillance (30). Data collected by U.S. SHIP or pre-movement testing programs could potentially inform EPS or vice versa. EPS could also support the NPB Pork Quality Assurance program, an initiative to help producers improve their production practices, through supporting visual inspections for disease (31). EPS could also collaborate with USDA's sick pig surveillance program by standardizing case definitions and connecting to findings from National Animal Health Laboratory Network laboratories. Biosecurity and movement data could be incorporated through the Rapid Access Biosecurity App, an application and service that helps standardize SPS biosecurity plans for producers and animal health officials (32). National swine disease monitoring efforts such as the Swine Disease Reporting System or MSHMP might also be collaborative with on-farm EPS. Precision farming tools, such as audio monitors for coughing and video cameras for huddling, could reduce staffing needs. However, these technologies are often expensive, require specific hardware, and are still under development. Despite this, participants felt these technologies should be explored for EPS.

Finally, participants identified many key challenges for EPS. Employee training and availability were major concerns. Participants reported that many employees have little or no background in swine production. Specific clinical signs, such as hemorrhagic diarrhea, would likely be too difficult for farm staff to identify. Training would need to be simple and accessible for those with different language or educational backgrounds. Otherwise, data quality would likely suffer. Additionally, many farms already experienced staff shortages for regular operations, and more surveillance could be burdensome. Maintaining participation in the absence of an ASF outbreak would also be difficult. While some early adopters would see value in supporting ASF preparedness, many would be hesitant because of a perceived low risk of an ASF introduction on their farm. Participants were also concerned about regulatory or government response to EPS suspect findings and thought that the potential for business disruptions during an FAD investigation would discourage reporting suspect cases in an ASF-free region. A regulatory framework to handle EPS suspects would be important. Participants also felt that some diagnostic testing would likely be necessary, but that prior to an ASF outbreak in the U.S., it would be difficult for farms to justify or afford this additional cost and time. Specimens that could be collected without opening up a carcass or validation of pen-level samples such as oral or processing fluids would help address these concerns. Data sharing and maintaining data privacy were another major challenge. To be effective, participants felt that some data or procedures may need to be communicated and shared between companies, but it would be difficult to coordinate. This would be especially challenging if data were shared with government, and many felt that some producers would not participate in government-led EPS.

## Discussion

This work explored current swine data collection and disease surveillance practices and private and public opportunities for enhancing ASF surveillance in the U.S. swine industry. Through the questionnaire and subsequent focus group discussion, many potential strengths and values of EPS protocols were identified, but many challenges and concerns were also recognized. While the questionnaire results indicate that disease surveillance practices are commonly conducted on U.S. swine farms, it is still unclear how much information reaches an electronic database, especially considering that nearly half of farms reported primarily collecting data through hand-written records. To improve data collection, new or existing technologies such as cell phones should be used directly in barns and pens by farm staff. Data could then be automatically uploaded to centralized management software. Management software usage was also high across all farm types, representing an opportunity to embed an EPS utility within the software. Alternatively, features like application programming interfaces (APIs) could centralize data from multiple software sources, so that data entered into a swine management system could potentially be automatically available for a surveillance application, or vice versa. APIs or other software connections are already used in the swine industry to link many types of software, such as for sharing movement or feed information. However, the type of software used by questionnaire respondents varied considerably, which may hinder the development of a uniform, data-monitoring EPS protocol. This view was repeated by participants in the world-café, who emphasized that standardized data collection will be critical for EPS protocols to be implemented across different software. Another technological consideration for EPS is to what extent it would rely on online or cellular access for functionality, as many farms are located in regions with limited connectivity.

Many opportunities for improvement and collaboration in disease surveillance were identified. High diversity in collected records suggests an opportunity to standardize disease data collection across the industry. Important signs of ASF including fever, skin discoloration, and huddling, were the least common to be recorded, but this may be improved through EPS protocols or precision farming technologies. Routine disease surveillance as described in the questionnaire might be an opportunity for implementing ASF surveillance with minimal extra cost to the producer through additional testing on suspect samples or multiplex assays. However, respondents rarely recorded test results into management software, though this may be more easily captured through collecting data directly from veterinary diagnostic laboratories. Notably, necropsies were not consistently performed on farms. In response to this questionnaire finding, participants from the world-café felt that necropsy and specimen collection procedures could be streamlined by not opening the carcass, developing techniques for easier collection of tissues such as lymph nodes, or by diagnostic testing of routinely collected oral or processing fluids. Necropsy findings are critical for surveillance, and improvements might be achieved through collaboration with programs such as the Certified Swine Sample Collector Training (33). EPS protocols should explore how these different specimens and testing schemes could be applied to maximize surveillance sensitivity and balance economic factors.

Some limitations were present in interpreting results from these activities. The questionnaire was only advertised to MN swine producers, and disease surveillance practices identified here might not be commonly shared throughout the U.S. Also, some important parts of the industry, such as small or show herd producers, were not represented in the world-café, so opportunities or challenges unique to these groups could not be collected in detail. This again highlights the need for improved ASF awareness and collaboration with these types of producers, as surveillance within these groups will be critical to protecting the U.S. swine industry. Despite these limitations, results from these activities have demonstrated a potential role for EPS to improve ASF early detection in the U.S. Future EPS protocols will need to be tested on swine farms to identify potential pitfalls in their application and fine-tune detection methods, and overall, any swine disease surveillance plan should be developed as a joint effort between researchers, industry, and, in case of ASF, government and regulatory officials. This work will help direct development of valuable EPS protocols for the U.S. swine industry.

## Data availability statement

The original contributions presented in the study are included in the article/Supplementary material, further inquiries can be directed to the corresponding author.

## Author contributions

WD conducted beta-testing and distributed the questionnaire. AP, RS, YC, and WD organized, moderated the focus group discussion, and summarized findings. RS analyzed the questionnaire data and wrote the manuscript. AP and YC edited it. All authors helped draft and revise the questionnaire. All authors contributed to the article and approved the submitted version.

## Funding

This work was funded by a cooperative agreement with the United States Department of Agriculture Center for Epidemiology and Animal Health. The findings and conclusions in this publication are those of the author(s) and should not be construed to represent any official USDA or U.S. Government determination or policy.

## Conflict of interest

The authors declare that the research was conducted in the absence of any commercial or financial relationships that could be construed as a potential conflict of interest.

## Publisher's note

All claims expressed in this article are solely those of the authors and do not necessarily represent those of their affiliated organizations, or those of the publisher, the editors and the reviewers. Any product that may be evaluated in this article, or claim that may be made by its manufacturer, is not guaranteed or endorsed by the publisher.

## Supplementary material

Supplementary File 1 Swine data collection and disease surveillance questionnaire.
