# Supplementary material for: Enhancing passive surveillance for African swine fever detection on U.S. swine farms
Source: Front Vet Sci. 2022 Dec 2;9:1080150. doi: 10.3389/fvets.2022.1080150 (PMC9755322; doi:10.3389/fvets.2022.1080150)
Supplement: Supplementary File 1 — Swine data collection and disease surveillance questionnaire. [file Data_Sheet_1.docx]

**Personalized Early Disease Warning System Questionnaire**

**UMN CAHFS**

**Introduction**

Welcome to the Personalized Early Disease Warning System survey!

From the team at the Center for Animal Health & Food Safety at the UMN, welcome and thank you for responding to this survey! Our goal is to identify how data you currently collect on your farm could be used for detecting a disease such as PRRS or a foreign animal disease outbreak such as African or Classical swine fever. We hope to create a tool that gives you added power to detect diseases quickly and efficiently on your farm using the software you already own.

**Instructions**

We ask that an owner or manager knowledgeable in data collection (particularly disease surveillance and data collection) complete this survey.

For producers that operate multiple types of farms where disease data collection differs significantly between sites, we ask that you have the manager of each farm type answer the survey individually.

For example, the manager of the sow unit would answer the survey for how data is collected on the sow farm, while the manager of the grow-finish unit would complete a separate survey for the grow-finish unit.

Eventually, there will be an opportunity for you to participate in testing our surveillance protocol. At the end of this survey, you may indicate whether you would like to be contacted about learning more about this project by filling out a separate contact form, keeping this survey anonymous. Indicating you want to be contacted will not require you to participate, nor will any contact information you provide be shared with any other group or agency. If you are more comfortable sending your contact information via email or want to hear more, you may email Dr. Rachel Schambow at scham083@umn.edu.

We appreciate your participation in our research. All of the information that you provide will be treated as confidential and will not be used to identify you in any way. This survey should take approximately 10-20 minutes to complete. Thank you!

1. What type of farm best describes this site?

- Farrow-to-finish
- Sow
  Gilt Development Unit
- Nursery
- Finisher
- Genetic multiplier
- Other: _______________

1. How many ***other*** sites share the same characteristics as this one? __________
2. Pig inventory on site:

- Sows: _______
- Nursing piglets: _______
- Adult boars: _______
- Adult gilts: _______
- Nursery pigs: _______
- Grow-finish pigs: _______

1. What is your role on this farm site?

- Owner
- Manager
- Farm staff
- Veterinarian
- Other (please specify): ____________

1. How often do you use a veterinary service?

- We have a veterinarian that makes regular visits to the farm.
- We have a veterinarian, but we only contact them for visits when we have a specific concern.
- We do not have a regular veterinarian but have access to veterinary services if needed.
- We have difficulty having access to veterinary services.

1. Please identify the digital software used for data collection and management on your farm:

- PigChamp
- MetaFarms
- Porcitec
- MTech
- Made by management company
- Made by veterinarian
- Other (please specify): ____________________
- We do not use software for data collection and management

1. Specify any outside source used to facilitate data recording and management:

- Management company
- Veterinarian
- No outside source used. Data collection and management are performed by farm staff.

1. Who primarily collects data prior to its entry into a management record or software?

- On-farm staff through hand-written records
- On-farm staff through handheld technologies (cell phone, data entry pad, other digital technology)
- Veterinarian or management representative
- Other (please specify): ____________________

1. Are you familiar with AgView?

- Yes, and one or more of my premises are enrolled
- Yes, but none of my premises are enrolled
- No, I am not familiar with AgView nor am I enrolled

1. Are you familiar with the Secure Pork Supply plan?

- Yes, and one or more of my premises has a plan
- Yes, but none of my premises has a plan
- No, I am not familiar with Secure Pork Supply nor have a plan in place

1. Are disease events recorded on the farm? (For example, respiratory, enteric, abortion)

- Yes
- No

*Logic Step: Questions 12-15 available if answer Yes to Question 11*

1. What level of detail are disease events recorded? Select all that apply. “Groups of animals” refers to data collected on a per pen, barn, or per other housing unit basis, rather than specifically for an individual pig.

- Individual animal, grow-finish pigs
- Individual animal, sows or boars
- Individual animal, nursery
- Groups of animals, sows or boars
- Groups of animals, grow-finish pigs
- Groups of animals, nursery
- Data collected on a whole-herd level (grow-finish)
- Data collected on a whole-herd level (sow or boar)
- Data collected on a whole-herd level (nursery)

1. What types of disease events are recorded?

- Sudden death
- Abortion
- Respiratory (cough, sneeze, trouble breathing, etc.)
- Enteric (diarrhea, vomiting, etc.)
- Off-feed/low feed intake
- Huddling
- Lameness or reluctance to move
- Fever
- Skin discoloration (red or blue areas)
- Other: ________________

1. Do you record a specific pathogen?

- Yes
- No

1. How frequently are disease events recorded?

- Daily
- Multiple days per week, but not daily
- Once per week
- Once per month
- No set schedule
- Other (please specify): ____________________

1. What types of production records are collected? Check all that apply.

- Mortality
- Pig weights and/or growth
- Carcass quality
- Feed consumption by pen
- Feed consumption by barn or premise
- Water consumption (any level)
- Treatment records (antibiotic usage or other veterinary care)
- Reason for why a sow was lost or removed from the herd
- Animal movements between pens or farms
- Movement of workers or equipment between farms
- Other: ________________________

1. What types of reproductive or breeding records are collected? Check all that apply.

- Breeding dates
- Pregnancy check results
- Rebreeding events
- Abortion dates
- Abortion cause
- Stillbirths
- Mummies
- Other: _____________

1. When would a drop in feed consumption be detected?

- Within hours
- Within a day
- Within a week

**Disease Surveillance - General**

1. How do you most commonly recognize when a disease event occurs?

- Inspection by farm staff
- Inspection by farm managers/owners
- Veterinarian inspection
- Other (please specify): ____________________

1. What factors trigger a thorough disease investigation? Check all that apply.

- Increased Death loss/mortality
- Increased illness/morbidity
- Water consumption
- Feed intake
- Gut feeling
- Other (specify): ____________

1. Do you feel that you would be able to recognize the signs of a possible FAD (Foreign Animal Disease) if one were introduced to your farm?

- Yes
- No
- Unsure

**Disease Surveillance – Sampling**

1. What diseases does your site routinely test for? Check all that apply.

- PRRS
- Influenza
- PEDV
- PDCoV
- TGEV
- Mycoplasma hyopneumoniae
- PCV2
- Other: _____________
- None

1. What type of live or necropsy sampling does your site conduct? Check all that apply.

- Blood
- Oral fluids
- Nasal swabs
- Tonsil
- Superficial inguinal lymph nodes
- Environmental or Swiffer sampling
- Processing fluids
- Airborne collection
- Other: _______________
- None

1. Are diagnostic test results reported into the on-farm management software or system?

- Yes
- No

1. Do you perform routine necropsies on the farm?

- Yes, by on-farm staff
- Yes, by veterinarian
- No, we do not perform routine necropsies

*Logic Step: Question 26 only available if answer Yes to question 25.*

1. If you or your veterinarian perform necropsies on-farm, how often do you perform them?

- All pigs that die on farm
- Most pigs that die on farm
- Infrequently or only on pigs that die of unknown causes
- Only in the event of a large-scale, on-farm disease outbreak
- We do not perform necropsies on-farm.

1. Would you or on-farm staff be able to necropsy a dead pig and collect samples?

- Yes, and we already perform these procedures on-farm
- Yes, we would be comfortable with these procedures, but do not regularly perform them.
- No, we would not feel comfortable now, but we might be with further training.
- No, and we would prefer that our veterinarian perform these procedures.

*Logic Step: Question 28 only available if answer Yes to question 27.*

1. Would you/on-farm staff feel comfortable collecting the following samples on necropsy?

- Spleen
- Tonsils
- Superficial lymph nodes
- We would be interested in additional training on sample collection

**Conclusion Page**

This concludes the data collection part of the survey. You may use the back arrows to review your answers before submitting. To submit your survey, make sure to click the button in the right-hand corner of your screen.

We will be looking for participants willing to test our enhanced passive surveillance protocol on their farm. Would you be willing to learn more about the enhanced passive surveillance program we are developing? To indicate your interest and to keep this current survey response anonymous, please go to this link to provide your name and contact information: https://umn.qualtrics.com/jfe/form/SV_9Ssx1n6C3udTG5M

Thank you!

**Follow-up Questionnaire Interest Form**

**Thank you for your interest in the Personalized Early Disease Warning System!**

As stated previously, we will be looking for participants willing to test our enhanced passive surveillance protocol on their farm. If you would be willing to learn more about the enhanced passive surveillance program we are developing, please list your name and email or phone number below.

If you want to learn more, you may email Dr. Rachel Schambow at scham083@umn.edu.

All contact information you provide will be treated confidentially.

______________________________________________________ [free entry box]
